# Omega-3 Fatty Acids and Alzheimer’s Disease: Toward a New Understanding of Neuroprotective Mechanisms and Intervention Strategies

**DOI:** 10.3390/md24070224

**Published:** 2026-06-25

**Authors:** Giacoma Galizzi

**Affiliations:** Institute for Biomedical Research and Innovation (IRIB), National Research Council (CNR), 90146 Palermo, Italy; giacoma.galizzi@irib.cnr.it

**Keywords:** Alzheimer’s disease, DHA, EPA, neuroinflammation, neurodegeneration, PUFA

## Abstract

Alzheimer’s disease (AD) is a multifactorial neurodegenerative disorder characterized by amyloid-β (Aβ) deposition, tau hyperphosphorylation, neuroinflammation, mitochondrial dysfunction, and oxidative stress. Despite recent advances, current therapies offer little benefit, and AD remains a significant challenge. Polyunsaturated fatty acids (PUFAs), particularly eicosapentaenoic acid (EPA) and docosahexaenoic acid (DHA), have attracted attention for their neuroprotective effects primarily through anti-inflammatory and antioxidant properties, but also for their ability to influence membrane fluidity and neuronal function. DHA is the predominant omega-3 PUFA in nerve cell membranes and is critical for synaptic plasticity and cognitive function. Some evidence has demonstrated that marine omega-3 supplementation reduces Aβ deposition, modulates microglial activation, and prevents cognitive decline in animal models. Even with heterogeneous results, preclinical and clinical studies suggest that long-term DHA/EPA supplementation can improve cognitive function in subjects with mild cognitive impairment (MCI) and reduce neuroinflammation markers. However, individual variability and brain bioavailability pose significant challenges. This review summarizes and discusses the current knowledge on the importance of PUFAs for human health, exploring novel mechanistic hypotheses, such as the effect of omega-3 fatty acids on brain iron homeostasis, the microbiota–gut–brain axis, the glymphatic system, and miRNAs. Furthermore, it focuses on the therapeutic potential of PUFAs in the treatment of AD and proposes future directions for translational research.

## 1. Introduction

Alzheimer’s disease (AD) is the most prevalent cause of dementia worldwide and constitutes one of the most significant challenges for modern healthcare systems due to its increasing global prevalence and the lack of effective disease-modifying therapies. Clinically, AD is characterized by a progressive deterioration of memory and cognitive abilities, accompanied by behavioral and neuropsychiatric disturbances that ultimately compromise functional independence [1]. At the neuropathological level, the disorder is traditionally defined by the accumulation of extracellular β-amyloid (Aβ) deposits and intracellular neurofibrillary tangles (NFTs) composed of hyperphosphorylated tau protein [1,2]. However, growing evidence indicates that AD pathogenesis involves a complex network of interacting mechanisms, including mitochondrial impairment, oxidative stress, metabolic dysregulation, and sustained neuroinflammatory responses, which, collectively, contribute to synaptic dysfunction, neuronal degeneration, and progressive brain atrophy [1,2,3].

Among the biological processes implicated in AD, alterations in lipid metabolism have recently emerged as an important component of the disease’s developmental progression [4,5]. The brain is particularly enriched in lipids that are essential for neuronal membrane organization, signal transduction, and synaptic communication. Disturbances in lipid homeostasis may therefore have profound consequences for neuronal stability and function. In this context, several studies have reported reduced concentrations of docosahexaenoic acid (DHA) in both the cerebral tissue and cerebrospinal fluid (CSF) of patients with AD [6]. Because DHA is a key structural and functional constituent of neuronal membranes, its depletion may negatively influence membrane fluidity, synaptic plasticity, and the capacity of neurons to adapt to metabolic and inflammatory stressors [7].

Polyunsaturated fatty acids (PUFAs) are a class of fatty acids characterized by two or more double bonds in the carbon chain, and they are widely recognized as being involved in numerous physiological processes. Marine-derived lipids are particularly rich in long-chain PUFAs belonging to the omega-3 and omega-6 series, which are essential for brain development and maintenance, contributing to membrane architecture, neuronal signaling, and inflammation modulation [8,9,10]. Among these, the long-chain omega-3 fatty acids DHA and EPA have attracted considerable interest due to their potential neuroprotective properties. Experimental studies suggest that these fatty acids can influence a range of biological mechanisms associated with neurodegeneration, including inflammatory signaling, oxidative balance, and synaptic function, showing neuroprotective and pro-cognitive effects [11].

DHA is especially abundant in neuronal phospholipid membranes, where it contributes to the maintenance of membrane fluidity and supports efficient synaptic transmission and receptor activity. EPA, although present in smaller amounts in brain tissue, appears to play an important regulatory role in systemic and central inflammatory responses [4]. Through these complementary actions, omega-3 PUFAs may influence multiple pathways involved in neuronal survival and brain homeostasis.

Over the past decades, an expanding body of research has explored the relationship between omega-3 fatty acids and cognitive health [5,6]. While several experimental studies have suggested beneficial effects on neurodegenerative processes, clinical findings remain doubtful and sometimes inconsistent. This body of data, sometimes conflicting, highlights the need for a clearer understanding of the mechanisms through which omega-3 PUFAs may influence the risk of AD onset and progression. At the same time, it has also paved the way for their potential use as an attractive therapeutic strategy to combat cognitive decline and the course of the disease.

In this context, this review aims to provide a comprehensive overview of current knowledge regarding the biological functions of omega-3 fatty acids and their potential involvement in the pathophysiology of AD, with a focus on recent advances, underexplored areas, and emerging trends in this field of research.

## 2. Microalgae as a Rich Source for PUFA

Marine algae and phytoplankton, along with fish such as salmon, mackerel, sardines, herring, and tuna, are the main natural sources of long-chain omega-3 polyunsaturated fatty acids [8,9].

Microalgae are a group of autotrophic microorganisms that live in marine, freshwater, and soil ecosystems [12]. Through photosynthesis, they produce various organic substances and bioactive compounds of high biological and nutritional value [13,14]. The market for microalgae-based pharmaceuticals and nutraceuticals is rapidly expanding thanks to their content rich in vitamins, minerals, PUFAs, carotenoids, phycobiliproteins, polysaccharides, phenolic compounds, and phycotoxins. *Spirulina platensis* [15,16], *Aphanizomenon flos-aquae* [17], *Dunaliella salina* [18], *Chlorella* sp. [19], and *Haematococcus pluvialis* [20] are some of the most widely used microalgae species, both for their high nutritional value and potential health benefits [21].

Microalgae are the main producers of marine PUFAs, and their average lipid content can reach up to 40%, depending on the species and growth conditions [9]. The most studied species are *Schizochytrium* sp. and *Crypthecodinium cohnii*, which are rich in DHA [22,23], and *Nannochloropsis* sp. and *Phaeodactylum tricornutum*, which are rich in EPA [24,25]. Brown and red algae also contain moderate amounts of EPA and DHA along with minor PUFAs such as stearidonic acid and γ-linolenic acid (GLA) [13], while Cyanobacteria such as *Spirulina platensis* and *Aphanizomenon flos-aquae* (AFA) contain mainly GLA and α-linolenic acid (ALA) [15].

## 3. A Look at the Main Characteristics of PUFA

### 3.1. Structure and Metabolism

Fatty acids are the fundamental structural components of many complex lipids and play a central role in cellular metabolism and membrane organization. Based on the degree of saturation of their hydrocarbon chain, fatty acids are generally classified into three major groups: saturated fatty acids (SFAs), monounsaturated fatty acids (MUFAs), and polyunsaturated fatty acids (PUFAs). SFAs lack carbon–carbon double bonds, whereas unsaturated fatty acids contain one (MUFAs) or more double bonds (PUFAs) along the carbon backbone. The classification of PUFAs into omega-3 (ω-3) or omega-6 (ω-6) families depends on the location of the first double bond relative to the methyl terminus of the molecule. In biological systems, fatty acids are mainly stored in the form of triglycerides, which function as energy reservoirs. Through the process of β-oxidation, these molecules are catabolized to generate metabolic energy that ultimately supports the synthesis of adenosine triphosphate (ATP) [26].

Among the omega-3 PUFAs, eicosapentaenoic acid (EPA; 20:5 n-3) and docosahexaenoic acid (DHA; 22:6 n-3) are two of the most biologically relevant species. Both are derived from the essential precursor α-linolenic acid (ALA; 18:3 n-3). Within the omega-6 family, arachidonic acid (AA; 20:4 n-6) is one of the most important metabolites produced from linoleic acid (LA; 18:2 n-6). Because humans lack the enzymatic machinery required for the de novo synthesis of ALA and LA and can convert ALA to EPA and DHA only in limited amounts, these fatty acids must be consumed through an appropriate diet to obtain health benefits [10,27,28]. Marine products such as microalgae and fish are among the best dietary sources of omega-3 PUFAs [8].

The capacity to synthesize fatty acids varies among tissues. While the central nervous system (CNS) is able to produce saturated and monounsaturated fatty acids, the biosynthesis of long-chain omega-3 and omega-6 PUFAs occurs predominantly in the liver. Nevertheless, additional metabolic activity has also been reported in other tissues, including the brain, skeletal muscle, testes, kidneys, and adipose tissue [28]. The conversion of essential fatty acids into long-chain PUFAs involves a coordinated series of enzymatic reactions mediated by fatty acid desaturases (FADS) and elongases (ELO). Enzymes belonging to the FADS family introduce additional double bonds into the fatty acid chain, whereas ELO enzymes extend the carbon backbone through the addition of two-carbon units [29].

Following their synthesis, PUFAs are transported from peripheral tissues to the brain via the bloodstream associated with albumin or lipoproteins. There, DHA can be present in an unesterified form or in a form bound to lysophosphatidylcholine (LysoPC-DHA) [3,4]. The mechanism by which fatty acids cross the blood–brain barrier (BBB) and enter the central nervous system (CNS) is still under investigation. The most widely accepted hypothesis combines passive diffusion, typically used by unbound fatty acids, and protein-mediated transport. Several studies support passive diffusion as the primary mechanism used by DHA and EPA to cross the BBB, due to their ability to undergo rapid flip-flop—transbilayer movement across endothelial cell membranes [30,31].

The absorption of esterified fatty acids, instead, involves specialized membrane proteins that facilitate their passage. It has been hypothesized that esterified omega-3 PUFAs are captured by endothelial cells through major facilitator superfamily domain-containing protein 2a (Mfsd2a), which is highly expressed in brain microvascular endothelial cells, and sequestered by transport carriers such as fatty acid-binding protein 5 (FABP5) to cross the BBB [32,33]. Finally, they pass through the endothelial basement membrane via specific fatty acid transport proteins (FATPs) that couple the import of fatty acids into cells with a long-chain acyl-CoA synthetase [34].

A study conducted by Ouellet et al. demonstrated that the absorption of DHA and EPA is not a saturable process at concentrations up to 100 µM, indicating that these compounds prefer passive diffusion to cross the BBB and enter the CNS [30]. Passive diffusion appears to play a significant role under conditions of high fatty acid concentrations. Conversely, when concentrations are low, protein-mediated transport becomes essential to maintain an adequate supply of fatty acids to the brain. Both systems are therefore essential for maintaining brain lipid homeostasis [35].

**Figure 1:**
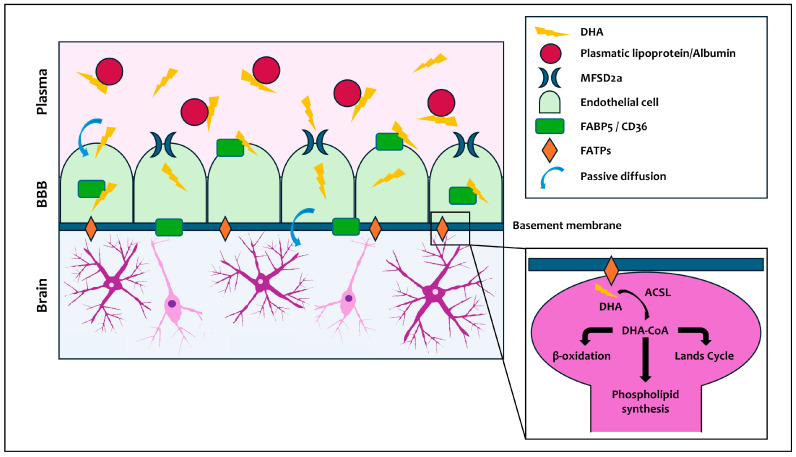
Schematic representation of the BBB with the blood capillary, neurons, and astrocytes end-feet. Polyunsaturated fatty acids, such as DHA, travel from the liver to the brain through the circulatory system, bound to albumin and circulating lipoproteins. They are captured by the MFSD2a protein in endothelial cells and sequestered by FABP5 to pass through the BBB. Finally, they cross the basement membrane via specific FATPs or passive diffusion. At the bottom right, a magnified view of the black rectangle illustrates the different fates of DHA once in the brain. DHA, converted to DHA-CoA by ACSL, can enter various metabolic pathways, including β-oxidation, recycling via the Lands cycle, and phospholipid synthesis pathways in the endoplasmic reticulum.

Once in the brain, fatty acids are rapidly activated by enzymes such as long-chain acyl-CoA synthetase (ACSL), generating acyl-CoA derivatives that can enter multiple metabolic pathways, including β-oxidation in mitochondria and peroxisomes, recycling via the Lands cycle, and phospholipid synthesis pathways in the endoplasmic reticulum, as summarized in Figure 1. Through these pathways, fatty acids contribute to the synthesis of several lipid classes, including diacylglycerols, triacylglycerols, phosphatidylethanolamine (PE), and phosphatidylcholine (PC), which are essential for membrane structure and cellular signaling [36,37]. In particular, the enrichment of neuronal membranes in long-chain PUFAs, especially DHA, is considered essential for maintaining membrane fluidity, synaptic function, and neuronal communication. Alterations in the availability or metabolism of these lipids can therefore affect brain physiology, and these have been increasingly implicated in the development of neurodegenerative diseases, including AD.

Among cytosolic fatty acid-binding proteins (FABPs), several isoforms are involved in intracellular transport and fatty acid metabolism. In the CNS, FABP5 plays a particularly important role in lipid signaling and neuronal physiology. This protein participates in multiple biological processes, including neurotransmission, neurogenesis, cognitive function, and the regulation of circadian rhythms [38,39]. FABP5 displays a high affinity for both AA and DHA, suggesting that it contributes to maintaining the balance between omega-6 and omega-3 PUFAs within neural tissues. Through this regulatory function, FABP5 may influence both inflammatory responses and mechanisms involved in tissue repair and regeneration [38].

Several authors have reported that, during development, different regions of the CNS, such as the cerebral cortex, hippocampus, cerebellum, spinal cord, and retina, present high levels of FABP5 [40,41,42]. These levels decrease during the early postnatal period and remain constant in the adult brain in both neurons and glia [43,44]. Interestingly, experimental studies using transgenic mouse models of AD (APPswe/PSEN1ΔE9) have reported a marked reduction of FABP5 expression in cerebral capillaries. This reduction was accompanied by decreased transport of radiolabeled DHA across the BBB, as well as increased susceptibility to cognitive impairment in animals fed diets deficient in omega-3 fatty acids [45]. Consistent with these findings, reduced levels of DHA in brain tissues have also been reported in patients affected by neurodegenerative disorders such as AD and Parkinson’s disease (PD).

Another key regulator of PUFA transport across the BBB is MFSD2a, which is highly expressed in the endothelial cells of brain microvessels. Animal studies have shown that MFSD2a-knockout mice exhibit significantly reduced levels of DHA in the brain, accompanied by neuronal loss in regions such as the hippocampus and cerebellum. These alterations are associated with behavioral abnormalities, including cognitive deficits and increased anxiety-like behavior [33].

From a biochemical perspective, PUFAs are distinguished by the presence of two or more double bonds within their long carbon chains. This structural feature confers a high degree of flexibility and fluidity to the lipid membranes in which they are incorporated. Because of these properties, PUFAs play an essential role in maintaining membrane dynamics and functionality [46]. In neuronal cells, membrane fluidity is particularly important for synaptic receptor activity, vesicle trafficking, and the processes of endocytosis and exocytosis. These membrane-related functions are closely associated with synaptic plasticity, a key mechanism underlying learning and memory, which is often impaired during the early stages of AD.

Beyond their structural role in membranes, PUFAs exert a wide range of biological effects. They act as important sources of metabolic energy, modulate synaptic signaling [47,48,49], and regulate gene expression through interactions with several transcription factors such as peroxisome proliferator-activated receptors (PPARs), nuclear factor κB (NF-κB), sterol regulatory element-binding proteins (SREBPs), liver X receptors (LXRs), Toll-like receptors, and transforming growth factor-β (TGF-β) signaling [50,51].

**Figure 2:**
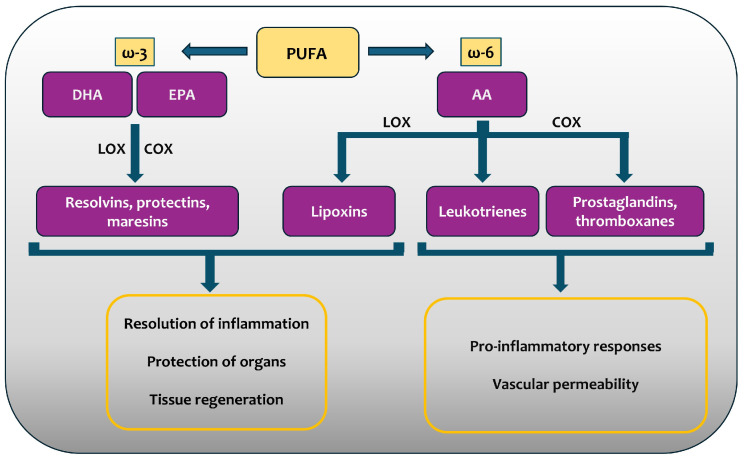
PUFAs are precursors of inflammatory signaling molecule mediators. DHA and EPA, derived from omega-3 fatty acids (ω-3), serve as precursors for the synthesis of SPMs. These include resolvins, protectins, and maresins, which participate in the resolution of inflammation, organ protection, and tissue regeneration. Omega-6 fatty acids (ω-6) are precursors of eicosanoids, such as leukotrienes, prostaglandins, and thromboxanes, inflammatory mediators formed from arachidonic acid (AA) via the lipoxygenase (LOX) and cyclooxygenase (COX) pathways, as well as lipoxins. They act on inflammatory responses and vascular homeostasis.

Furthermore, PUFAs serve as precursors for numerous bioactive lipid mediators involved in the regulation of inflammatory responses. Particularly, omega-6 fatty acids can be metabolized into eicosanoids derived from AA, including leukotrienes, prostaglandins, and thromboxanes. These molecules are involved in inflammatory processes and have been implicated in the pathogenesis of several disorders, such as atherosclerosis, bronchiolitis obliterans, and metabolic diseases [52,53,54]. In contrast, omega-3 fatty acids generally give rise to lipid mediators with anti-inflammatory and pro-resolving properties, and they have been associated with beneficial effects in conditions such as cardiovascular disease, bronchial asthma, cancer, diabetes mellitus, and metabolic syndrome [52,54,55,56,57,58]. EPA and DHA can be enzymatically converted into a family of a particular class of bioactive compounds collectively known as specialized pro-resolving mediators (SPMs). This group includes resolvins, protectins, and maresins, which actively contribute to the resolution of inflammatory responses, promote tissue repair, and support the restoration of physiological homeostasis (Figure 2) [59,60]. For this reason, maintaining an appropriate dietary balance between omega-3 and omega-6 PUFAs is considered essential for preserving metabolic health and preventing chronic inflammatory conditions.

In summary, PUFAs play a fundamental role in neuronal physiology by contributing to membrane architecture, energy metabolism, inflammatory regulation, and lipid-mediated signaling pathways. Their ability to influence membrane fluidity, synaptic transmission, and gene expression highlights their importance in maintaining normal brain function. Consequently, alterations in PUFA availability, transport, or metabolism may significantly affect neuronal homeostasis, and these have been increasingly implicated in the development of neurodegenerative disorders. Growing evidence suggests that imbalances in omega-3 and omega-6 fatty acids may affect pathological processes associated with AD. For this reason, understanding the specific mechanisms through which omega-3 PUFAs influence brain function has become an important area of research in the context of neurodegeneration.

### 3.2. The Role of PUFAs in the Human Brain

The human brain has a very high lipid composition, so fatty acids play an essential role in brain function. In recent years, numerous sources of published data have shown PUFA involvement in brain development [61,62], synaptic transmission [47,63], glucose uptake, and brain energy metabolism [64,65]. Moreover, PUFAs participate in neuroprotective, neuroinflammatory, and neurotrophic processes [66], thus maintaining cerebral and retinal function [67].

Omega-3 and omega-6 PUFAs are, in fact, the main constituents of the cell membrane and, thanks to their chemical characteristics, influence its fluidity, structure, and functions. They are also present in myelin and organelles in the form of PC, PE, and phosphatidylserine (PS) [68,69]. They, therefore, support the CNS structure, participating in the development and function of neurons, synapses, and endothelial cells and playing a pivotal role in cognitive events such as learning and memory [66].

Several studies support the role of omega-3 PUFAs in promoting synapse formation and the maturation and enhancement of synaptic transmission, which is essential for the propagation of neuronal information. Numerous mechanisms are involved in this process. Specifically, DHA promotes neurite outgrowth and synaptogenesis [61,70]. Furthermore, it is involved in the composition of synaptic membrane phospholipids, enhancing the levels of PS and phosphatidylinositol (PI), which are implicated in the release of synaptic vesicles [69]. Additionally, it promotes synaptic structure and function by increasing the expression of postsynaptic density protein 95 (PSD-95) and synapsin-1 and raising the level of drebrin, a dendritic spine protein crucial for synaptic plasticity [71,72]. These events are vital for learning and memory consolidation.

Interesting studies by Wu et al. have shown that DHA can re-establish synaptic connections and preserve cognitive ability lost following brain trauma [73,74]. These results have recently been confirmed by Lau et al. [75].

Studies using several models have shown that a diet poor in omega-3 PUFAs is associated with reduced DHA levels in neuronal cell membrane phospholipids, decreased cognitive function, and memory deficits [76,77], likely due to DHA’s role in modulating neurotransmission and synaptic signaling.

An adequate intake of omega-3 PUFAs, particularly DHA, during gestation and in the first months of life supports the neurocognitive development in newborns [34,78].

Dietary omega-3 fatty acid deficiency and brain AA/DHA imbalance during perinatal or postnatal development in mouse models are associated with synaptic impairment [58], reduced neurogenesis [79,80], altered brain glucose uptake and metabolism [64], cognitive deficits [81], and behavioral abnormalities such as depression and aggression [82,83].

Similarly, low DHA levels caused by the impairment of systemic DHA synthesis can modify the expression of neuronal plasticity and inflammation markers in the mouse brain [84]. Conversely, dietary DHA supplementation improved neurogenesis and synaptic transmission [61].

Age-related cognitive decline also appears to be linked to PUFA levels. Some studies report an association between reduced circulating EPA [85] or DHA [86,87] levels in older adults and an increased risk of developing dementia. DHA supplementation improves memory and cognitive activity in older healthy subjects [88].

Furthermore, interestingly, PUFAs have been shown to be highly promising in counteracting cognitive decline related to AD. Several authors have reported an association between low PUFA levels in CSF and plasma in AD patients with a higher risk of cognitive deficit. DHA supplementation has shown a significant improvement in cognitive functions compared to placebo [89] and could reduce the risk of disease onset [90]. The important role of PUFAs in neurocognitive development and in maintaining proper brain health is therefore evident.

## 4. Key Neuroprotective Effects of DHA: A Strategic Role for AD Treatment

Given the critical role of omega-3 PUFAs in maintaining neuronal structure and regulating inflammatory responses, increasing attention has been directed toward their potential involvement in the pathophysiology of AD.

AD is a neurodegenerative disease with a multifactorial etiology, generally associated with aging, which leads to a progressive deterioration of neuronal cells and loss of cognitive functions [1]. At the molecular level, AD is characterized by the massive presence of the hyperphosphorylated microtubule-associated protein tau [91,92] and extracellular plaques of Aβ that form from the cleavage of amyloid precursor protein (APP) by beta and gamma secretases [93,94]. These events are thought to cause cytoskeletal disorganization and to disrupt physiological cellular communication, contributing to neuroinflammation and neuronal damage [3,95].

Despite numerous studies, the exact etiopathogenesis of AD remains unclear. Until now, most therapeutic strategies have targeted Aβ accumulations, although in recent years, other factors responsible for the disease have emerged, such as neuroinflammation [96], oxidative stress [97], mitochondrial dysfunction [2], brain iron accumulation [98,99], cerebral glucose hypometabolism [100,101], and insulin resistance (IR) [102]. Recently, advances have been made in both the study of Aβ metabolism and understanding its clearance mechanisms. Much attention has been focused on the glymphatic system, the perivascular drainage pathways responsible for eliminating waste products from the brain [103].

Despite these advances in understanding pathological mechanisms, AD still poses a major challenge for biomedical research, and to date, no clinical therapies capable of modifying the course of the disease have been developed. Given the complexity of the disease, simultaneously targeting multiple factors can be a winning strategy. Omega-3 fatty acids have demonstrated a wide range of neuroprotective effects, making them a promising adjunct in the fight against AD.

Below, we analyze the various neuroprotective mechanisms of PUFAs, highlighting novel pathways that should be further explored to provide a protective strategy against AD and age-related cognitive deficits.

### 4.1. Omega-3 PUFAs as Regulators of Aβ and Hyperphosphorylated Tau Production

Amyloid plaques are extracellular deposits of Aβ peptides that arise from the aberrant proteolytic processing of APP by β- and γ-secretases [104,105]. Together with NFTs composed of hyperphosphorylated microtubule-associated protein tau, these aggregates are the principal neuropathological hallmarks of AD. The accumulation of Aβ in the extracellular space surrounding neurons is thought to initiate a cascade of pathological events, including oxidative stress, mitochondrial dysfunction, neuroinflammatory responses, and disturbances in brain insulin signaling, all of which contribute to progressive neuronal damage. In addition to their neurotoxic effects, both Aβ aggregates and pathological tau modifications interfere with synaptic function and plasticity, ultimately impairing neuronal communication and accelerating cognitive decline [106,107,108].

Several studies have demonstrated that adequate dietary intake of DHA may modulate key pathological processes associated with AD. Indeed, DHA appears to influence multiple steps involved in Aβ homeostasis. On the one hand, it reduces Aβ production by downregulating the activity of enzymes implicated in amyloidogenic processing, including presenilin-1 and beta-secretase (BACE1) [109,110,111]. On the other hand, DHA promotes Aβ clearance through several mechanisms, such as enhancing the activity of insulin-degrading enzyme (IDE) [112] and improving the function of the brain glymphatic system via the regulation of aquaporin-4 (AQP4), the most abundant water channel in the CNS [113]. Furthermore, as proposed by Grimm et al., DHA reduces the amyloidogenic processing of APP and increases the non-amyloidogenic pathway [114]. Moreover, its bioactive derivative neuroprotectin D1 further contributes to neuroprotection by reducing Aβ levels and enhancing neuronal survival in AD experimental models by modulating BACE1 expression and activity through the activation of peroxisome proliferator-activated receptor gamma (PPARγ) [115].

Consistent with these observations, a study by Oksman et al. demonstrated that supplementation with DHA significantly reduced the production of Aβ in APPswe/PS1dE9 transgenic mouse models of AD [116].

In addition to its effects on amyloid pathology, DHA has also been shown to attenuate aberrant phosphorylation of the tau protein. This ability appears to be mediated, at least in part, by the inhibition of key kinases implicated in tau hyperphosphorylation, including glycogen synthase kinase-3 beta (GSK-3β) and c-Jun N-terminal kinase (JNK), thereby contributing to the modulation of tau-related neurodegenerative processes [117,118,119].

Collectively, these findings suggest that DHA may limit Aβ generation and accumulation as well as tau hyperphosphorylation through the coordinated regulation of multiple molecular pathways.

### 4.2. The Key Role of Omega-3 PUFAs in Neuroinflammation

Chronic neuroinflammation is a hallmark of pathological conditions such as aging or AD and participates in the neurodegenerative process [120,121].

Accumulating evidence indicates that Aβ peptides can activate microglial cells, promoting the release of pro-inflammatory cytokines such as tumor necrosis factor alpha (TNFα) and interleukin-1 beta (IL-1β) [122], thereby sustaining a chronic inflammatory environment in the brain. In this context, increasing attention has been directed toward the anti-inflammatory properties of omega-3 PUFAs, particularly DHA.

Both in vivo and in vitro studies suggest that DHA can modulate microglial activation by promoting a shift toward the anti-inflammatory M2 phenotype, partly through the inhibition of NF-κB nuclear translocation via the Sirtuin 1 (SIRT1) signaling pathway [123,124].

Additional mechanisms have been proposed for the anti-inflammatory activity of DHA, including the activation of PPARγ [125] and the inhibition of the P38 mitogen-activated protein kinase (MAPK) signaling pathway [126]. In addition to its anti-inflammatory activity, DHA has also been shown to promote microglial phagocytosis of Aβ aggregates [127].

Consistent with these findings, a study by Chang et al. has shown the preventive action of DHA on LPS-induced neuroinflammation, associated with a recovery of synaptic structure and function in CA1 pyramidal neurons of the hippocampus [128].

García-Domínguez et al. [129] and Charrière et al. [130] have recently summarized in vitro and in vivo studies on the anti-inflammatory activity of PUFAs in neurodegenerative disease.

Furthermore, in the brain, omega-3 PUFAs may also exert indirect neuroprotective action through the synthesis of bioactive derivatives. As previously mentioned, DHA and EPA are, in fact, precursors of important SPMs with anti-inflammatory and pro-resolution actions such as the E-series resolvins (RvE1-E3), derived from EPA, and the D-series resolvins (RvD1-D6), maresins (Mar1-2), and neuroprotectins (NPD1), derived from DHA [60].

Resolvins (Rvs) act through G protein-coupled receptors (GPCRs) [131]. E-series resolvins, through their binding to the Chemokine-like Receptor (ChemR23) localized in microglia and neurons of the prefrontal cortex and hippocampus, increase the phagocytosis of apoptotic cells, decrease pro-inflammatory cytokine levels, and simultaneously induce beneficial resolution signals [132,133].

Similarly, D-series resolvins reduce the production of pro-inflammatory cytokines by regulating specific micro-RNAs (miRNAs) and inhibiting NF-κB activation [134,135,136]. Particularly, RvD1 has been shown to promote the transition of microglial cells towards the IL-4-induced protective/pro-resolution phenotype through the pro-inflammatory PPARγ signaling pathways and to enhance the phagocytosis of Aβ [137,138].

Bathina et al. suggest that the anti-inflammatory and neuroprotective action of RvD1 may involve the modulation of the PI3K/Akt/mTOR pathway in the brains of diabetic mice [139].

The DHA-derived mediators neuroprotectin D1 (NPD1) and maresins also contribute to the resolution of neuroinflammation by regulating the levels of pro-inflammatory molecules through the inhibition of COX-2 and NF-κB expression and promoting tissue repair and neuronal survival [127,140].

Recently, Yin et al. showed that MaR1 treatment significantly improved cognitive decline, attenuated neuroinflammation, reduced Aβ42-induced production of the pro-inflammatory cytokines TNF-α, IL-6, and MCP-1, and increased levels of the anti-inflammatory cytokines IL-2 and IL-10 in a mouse model of AD [141]. Furthermore, MaR1 treatment upregulated the levels of PI3K/AKT and survival-related ERK, and downregulated the levels of proteins associated with inflammation, autophagy, and apoptosis, such as p38, mTOR, and caspase-3 [141]. The neuroprotective activity of MaR1 treatment was later confirmed by Shrivastava et al., who showed improvement in AD symptoms after long-term treatment of transgenic 5xFAD mice with MaR1 [142].

Interestingly, the levels of the resolvins RvD4, RvD1, RvE4, neuroprotectin NPD1, and the maresin MaR1 were found to be decreased in subjects with AD and MCI compared to controls, and, consistently, pro-inflammatory mediators were higher in AD and MCI [142,143].

Furthermore, recent studies have shown a significant increase in ChemR23 receptors in several postmortem human brain regions from AD, early-onset AD, and MCI patients compared to controls, suggesting the dysregulation of inflammation-resolution pathways during disease progression [144,145].

In line with these findings, dietary supplementation with DHA-rich formulations in individuals with AD or MCI has been associated with reduced pro-inflammatory cytokine release, increased resolvin D1 levels, inflammatory genes restored to physiological levels, and enhanced Aβ phagocytosis [144,145,146,147,148,149,150,151,152].

Likewise, aged mice fed fish-oil-enriched diets for 2 months showed increased brain DHA levels, accompanied by reduced expression of proinflammatory cytokines, improved morphological changes of astrocytes in the hippocampus, and restored memory deficits [153].

Taken together, these observations highlight the key role of DHA and/or its bioactive derivatives in modulating neuroinflammatory pathways and suggest that omega-3 PUFAs may represent a promising strategy for the complex management of neurodegenerative diseases such as AD.

### 4.3. Omega-3 PUFAs, Allies Against Oxidative Stress

Deposits of Aβ peptide and hyperphosphorylated tau proteins in the nervous system, along with the chronic inflammation present in the brain of AD patients, are associated with high levels of oxidative stress, which is characterized by an increase in reactive oxygen species (ROS) and a reduction in antioxidant systems [154].

An excess of ROS can cause changes in macromolecules such as DNA, proteins, lipids, and carbohydrates, affecting membrane peroxidation and contributing to the neuronal damage that occurs in aging and neurodegenerative diseases [155]. In turn, oxidative stress can also trigger an inflammatory response and increase the expression of BACE1 and the generation of Aβ plaques [156].

Postmortem studies on the brains of AD patients have shown increased markers of oxidative damage [157,158], while dietary DHA supplementation reduced levels of oxidative stress in the hippocampus [159,160]. Several studies have been conducted both in vitro and in vivo on the antioxidant effects of DHA [161,162,163,164,165], and these are summarized in the recent review by Borgonovi et al. [166].

Among the mechanisms proposed to explain the antioxidant action of omega-3 PUFA, the capacity of DHA to activate the Nrf2/Keap1/ARE gene expression pathway stands out. This pathway regulates the gene transcription of several antioxidant enzymes, including heme oxygenase (HO-1), glutathione peroxidase 4 (GPX4), nicotinamide adenine dinucleotide (NAD), and quinone oxidoreductase 1 (Nqo1) [167,168,169,170,171,172].

Furthermore, thanks to their ability to modulate glial cells and induce microglial polarization in the M2 phenotype, omega-3 fatty acids reduce the production of NO and ROS, which are responsible for oxidative stress [173].

Similarly, active SPM metabolites also have a strong antioxidant action. Resolvins modulate ROS production through PKA-mediated inhibition of the NADPH oxidase NOX2 [174,175]. NDP1 activates cell survival mechanisms and constrains oxidative stress-induced apoptosis by upregulating the anti-apoptotic proteins Bcl-2 and Bcl-xL, reducing the expression of the proapoptotic proteins Bax and Bad, and inhibiting the activation of caspase-3 [176]. Halapin et al. proposed that NDP1 promotes cell integrity and survival upon exposure to oxidative stress by enhancing the activation of the PI3K/Akt pathway [177,178]. Sun et al. instead have shown the ability of maresin to exert an antioxidant effect by regulating the Nrf-2/HO-1 pathway [179].

In short, these findings suggest that supplementation with omega-3 PUFAs may be a promising strategy to counteract the neurodegenerative processes associated with AD. Their combined antioxidant, anti-inflammatory, and anti-amyloidogenic properties could contribute to the modulation of key molecular pathways involved in neuronal damage and disease progression.

### 4.4. Omega-3 PUFAs in Ferroptosis: Friends or Foes?

Ferroptosis is a type of oxidative stress characterized by the peroxidation of plasma membrane phospholipids. This leads to programmed cell death induced by iron accumulation under conditions of reduced activity of GPX4, the main enzyme capable of reducing oxidized membrane phospholipids [180,181].

In recent years, several studies have demonstrated the presence of iron accumulation and lipid peroxidation in the early stages of AD [182,183,184]. Furthermore, in human brains affected by AD, GPX4 is downregulated in an early phase of the disease, whereas it is upregulated in a later phase, suggesting a compensatory mechanism in which cells increase transcription to compensate for reduced GPX4 protein levels [184].

Interestingly, ferroptosis markers, including iron dyshomeostasis [185], elevated ROS and lipid peroxidation levels, and reduced GSH and GPX4 antioxidant systems, have been observed in models of neurodegenerative diseases such as AD and PD, strengthening the hypothesis of the involvement of ferroptosis in disease’s pathogenesis [186,187,188,189].

An interesting review by Zhou et al. summarizes the knowledge acquired so far regarding the role of ferroptosis in the pathogenesis of AD [190].

PUFAs, including DHA, AA, and LA, are crucial components of cell membrane phospholipids and are greatly vulnerable to lipid peroxidation. Therefore, while on the one hand they are the main fuel for ferroptosis, on the other hand, as discussed previously, they show significant antioxidant activity. Several studies have highlighted DHA’s ability to enhance the antioxidant defenses of nerve cells, including the upregulation of the *GPX4* gene, protecting cell membranes against lipid peroxidation and playing a major role in inhibiting ferroptosis [190,191].

Moreover, as previously seen, DHA and its metabolite maresin-1 can counteract oxidative damage through the Nrf2-GPX4 pathway, suggesting their possible role in inhibiting neuronal ferroptosis in the cortex and hippocampus of mice and improving cognitive functioning [160,179,192].

The involvement of ferroptosis in neurodegenerative diseases such as AD and possible strategies to counteract them are an active area of investigation. Studying the action of omega-3 fatty acid metabolites in the prevention or blocking of neuronal ferroptosis may provide new insights and strategies for the treatment of AD.

### 4.5. The Protective Role of DHA in Mitochondrial Dysfunction

Mitochondrial dysfunction is considered a hallmark of aging as well as the main neurodegenerative diseases, including AD [193,194]. Elevated levels of intracellular Aβ can trigger mitochondrial dysfunction and lead to cell death through its ability to bind mitochondrial amyloid-binding alcohol dehydrogenase (ABAD), inhibit complex IV of the mitochondrial respiratory chain, increase ROS production, and alter mitochondrial dynamics [195].

Mitochondria are a major source of ROS and are particularly vulnerable to oxidative stress. This event, once triggered, creates a vicious cycle of ROS and mitochondrial dysregulation that culminates in cell death by apoptosis [196]. The high levels of ROS that characterize the oxidative stress process can damage mitochondrial function by inhibiting the mitochondrial respiratory chain, altering membrane permeability, and unbalancing Ca2^+^ homeostasis. Furthermore, they can induce mitochondrial DNA mutations and changes in the gene expression profile [196].

Numerous studies have, therefore, examined the action of various antioxidants and ROS-scavenging compounds on neurodegeneration and analyzed the impact of omega-3 fatty acids in controlling oxidative stress and restoring mitochondrial function [197,198].

A study conducted by Li et al. on HepG2 cells demonstrated the antioxidant activity of DHA but also showed its ability to promote mitochondrial transcription and enhance mitochondrial biogenesis. These effects were associated with the increased mRNA expression of key mitochondrial transcription factors, including mitochondrial transcription factor A (TFAM), mitochondrial transcription factor B2 (TFB2M), nuclear respiratory factor 1 (Nrf1), estrogen-related receptor alpha (ERRα), and PPARG coactivator 1 alpha (PGC-1α) [199]. These results are consistent with previous work by Lee et al. on C2C12 muscle cells in which both EPA and DHA modulated mitochondrial biogenesis and significantly increased PGC-1α, Nrf1, and TFAM mRNA levels and mtDNA copy number [200].

Additionally, studies by Busquets-Cortés et al. have shown DHA’s ability to influence mitochondrial dynamics in immune cells. DHA supplementation appears to favor the fusion process by increasing the expression of the mitofusin 1 and 2 proteins (Mfn-1 and Mfn-2) and optic atrophy protein 1 (Opa-1) [201]. In agreement with the role of DHA in mitochondrial dynamics, Zhang et al. have shown an improvement in the balance between mitochondrial fission and fusion and a weakening of oxidative stress in damaged cortical neuronal cells treated with DHA [202].

Consistent with these data, Park et al. demonstrated the protective role of DHA treatment on mitochondrial dysfunction in both the HT22 hippocampal cell line and the 5XFAD transgenic model of AD. In both models, DHA ameliorated the Aβ-induced impairment of mitochondrial respiration and mitochondrial dynamics [203].

Mitophagy was also enhanced by DHA treatment. In vivo and in vitro studies on ischemic stroke models showed that DHA protected neurons by eliminating damaged mitochondria through Pink1/Parkin-mediated mitophagy, reducing oxidative stress, and alleviating mitochondrial damage [204].

In this context, it is worth considering another important aspect of the neuroprotective process performed by omega-3 fatty acids, namely their anti-apoptotic activity. NPD1 helps counteract apoptotic signals and maintain mitochondrial integrity, especially under conditions of oxidative stress, by regulating mitochondrial apoptotic signaling pathways and modulating the activity and expression of Bcl-2 family members [176,205].

In murine models, the immediate administration of NPD1 after ischemic injury reduced mitochondrial-mediated apoptosis and protected mice from brain damage by blocking the translocation of BAX from the cytosol to the mitochondria and reducing caspase-3 activation [205].

The anti-apoptotic activity of DHA and its derivative NDP1 has also been demonstrated on human retinal pigment epithelial cells. NPD1 protects cells from oxidative stress-induced caspase-3 activation by positively regulating the anti-apoptotic proteins Bcl-2 and Bcl-xL and reducing the pro-apoptotic expression of Bax and Bad [176].

Exploring the protective role of omega-3 fatty acids against mitochondrial damage may pave the way for interesting therapeutic strategies for the treatment of various neurodegenerative diseases.

### 4.6. Effects of DHA on IR and Cerebral Glucose Metabolism

The brain is an insulin-sensitive organ. Through its receptor, insulin initiates a signaling cascade that regulates various neuronal functions and metabolic processes, including the translocation of glucose transporter 4 (GLUT-4) to the cell surface for glucose uptake [206]. An alteration of this pathway leads to IR that can damage the brain’s insulin signaling, with consequences that include impaired neuronal glucose uptake and metabolism, and metabolic dysfunction [207,208].

Insulin action in the brain is progressively impaired during Alzheimer’s-like neurodegeneration, and in recent years, a large body of data has suggested a link between metabolic disorders and AD due to a common state of IR and mitochondrial dysfunction [209,210,211,212].

Growing evidence has shown the ability of DHA to reduce peripheral IR through the activation of SIRT1 and the IRS/PI3K/AKT signaling pathway [213,214,215,216,217].

Aged rats fed a high-fat diet treated with DHA showed higher hippocampal insulin sensitivity and an improvement of cognitive function along with reductions in inflammation, oxidative stress, and Aβ and hyperphosphorylated tau accumulation [161].

A recent study by Thota et al. reported that dietary supplementation with DHA-rich fish oil conferred improved insulin sensitivity to individuals at high risk for IR and hyperinsulinemia, an effect associated with reduced plasma levels of GSK-3β [117]. Because GSK-3β is implicated in both the negative regulation of various aspects of insulin signaling and the pathogenesis of AD [218], these findings further support the potential role of omega-3 fatty acids as modulators of metabolic and neurodegenerative pathways relevant to AD [117,218].

Several studies have suggested that alterations in brain glucose metabolism are a feature of aging and may play an important role in the pathogenesis of AD [219].

Subjects with various neurodegenerative diseases, including AD, show a chronic state of cerebral glucose hypometabolism already in the preclinical stages of the disease [220,221,222], together with a reduced expression of the glucose transporters GLUT-1 and GLUT-3 [223,224].

Interestingly, various data sources have shown that dietary omega-3 fatty acids improve brain glucose metabolism [225,226]. A diet deficient in omega-3 PUFAs has been associated with reduced GLUT-1 levels in the rat cerebral cortex [64,65]. DHA supplementation increased GLUT-1 expression. Together, these data suggest a regulatory role for DHA in brain glucose transport and metabolism [227].

### 4.7. Omega-3 Fatty Acids Influence Cerebral Clearance Systems

The glia-lymphatic (glymphatic) system and the meningeal lymphatic system are the two cerebral clearance pathways linked to CSF flow that can facilitate the elimination of metabolic waste products, including Aβ and tau proteins [228]. Thus, they play a crucial role in maintaining cerebral homeostasis [229,230].

Growing evidence shows significant dysfunction in these systems with age, associated with reduced CSF clearance and the aggravation of age-related neurodegenerative processes [231,232].

The impairment of the glymphatic system has, in fact, been associated with the accumulation of Aβ [233,234] and phosphorylated tau [235]. The astroglial water channel AQP4 appears to play an essential role in the efficiency of this system [236]. In a recent study, Harrison et al. showed that the suppression of AQP4 significantly reduced tau clearance [237].

Furthermore, with the progression of AD, the efficiency of the glymphatic system decreases [238], and a reduction in the localization of AQP4 in the perivascular area can be observed, together with an increase in Aβ levels and a decline in cognitive function [239]. Consistently, in postmortem brains, Zeppenfeld et al. observed a significant reduction in the perivascular localization of AQP4, which was associated with worsening neurofibrillary pathology and Aβ levels [240].

Taken together, these data suggest that AQP4 may represent a promising pharmacological target for the regulation of the glymphatic system, making it a promising therapeutic strategy for AD.

Interestingly, the administration of omega-3 PUFAs in the form of fish oil enhanced interstitial clearance of Aβ from the brain in several mouse models, and this action was AQP4-dependent [113,241]. A very recent study by Cao et al. confirms the role of omega-3 PUFAs in enhancing the glymphatic system-mediated clearance of phosphorylated tau by restoring the reduced polarization of AQP4 via PDGF-B/PDGFRβ signaling and reversing sevoflurane-induced cognitive and motor deficits in neonatal mice [242].

Similarly, dysfunction in the meningeal lymphatic system contributes to the pathogenesis of age-related brain diseases [243]. Liu et al. demonstrated that long-term supplementation of omega-3 PUFAs can delay brain aging through meningeal lymphatic modulation, reduce the accumulation of phosphorylated tau, Aβ, and toxic metabolites in the brains of aged mice, and improve cognitive and motor functions [244].

Among the mechanisms involved in Aβ clearance from the brain to the blood, transport across the BBB plays a significant role. The major BBB efflux transporter is lipoprotein receptor-related protein 1 (LRP-1), whose expression decreases with aging and in AD patients [245,246]. Using APP/PS1 transgenic mice, Yan et al. showed a decrease in LRP-1 starting at 4 months of age, suggesting impaired BBB transport function at a very early stage of the disease. Interestingly, supplementation with fish oil rich in omega-3 PUFAs significantly increased LRP-1 expression levels, promoted Aβ clearance, and improved neuroinflammation levels [247].

This relatively new field of research offers an innovative approach to the treatment of age-related diseases. Therefore, a deeper understanding of the mechanisms underlying these clearance systems could lead to the identification of new potential therapeutic targets and open the way for innovative strategies to combat neurodegenerative diseases and promote healthy aging.

### 4.8. Omega-3 Fatty Acids and the Microbiota–Gut–Brain Axis

The CNS continuously and reciprocally exchanges information with the gut through various neuronal, metabolic, endocrine, and immune factors that travel through different metabolic pathways. Collectively, these are referred to as the “microbiota–gut–brain axis” [248,249,250].

These pathways include the immune system, the vagus nerve, tryptophan metabolism, the enteric nervous system (ENS), the neuroendocrine system with the hypothalamic–pituitary–adrenal (HPA) axis, and the circulatory system [248,250].

Human guts harbor a complex microbial community, known as the gut microbiota, which contributes to host physiology through the production of short-chain fatty acids (SCFAs) such as butyrate, propionate, and acetate. These metabolites play a critical role in maintaining microbial homeostasis, supporting the structural integrity of the intestinal epithelium, modulating epithelial energy metabolism, and regulating immune signaling pathways [251,252]. Furthermore, in recent years, several studies have demonstrated the ability of intestinal microbiota to modulate brain development and support CNS activity through their ability to produce neurotransmitters, including serotonin [253,254], dopamine [255], and γ-aminobutyric acid (GABA) [256,257,258,259].

A substantial body of data has established that the bacteria making up the microbiota essentially belong to two large phyla, Bacteroidetes and Firmicutes, which include different phylotypes at the species level [260]. Microbiota dysbiosis can induce augmented intestinal permeability and higher production of immunogenic cytokines and endotoxins such as TNF-α, IL-1β, and LPS that translocate into the bloodstream, triggering an inflammatory response that propagates from the periphery to the CNS [261,262]. Several studies on animal models have shown that gut microbiota dysbiosis can trigger neurodegeneration by modulating the neuroinflammatory process and microglia activation.

Furthermore, mice with dysbiosis or lacking microbiota have a compromised BBB and reduced hippocampal neurogenesis, which impairs memory and cognitive function [263,264,265].

Evidence from the literature has suggested that intestinal epithelial barrier dysfunction may contribute to systemic inflammation. Peripheral activation of microglia and monocytes can cross the BBB, promoting the release of pro-inflammatory cytokines and neurotoxic molecules. These mediators impair the clearance of Aβ and trigger neuroinflammatory responses, thereby contributing to the pathogenesis of AD [266,267,268].

In this regard, a growing body of data shows the involvement of the gut microbiome in neurodegenerative diseases [269,270]. A comprehensive and exhaustive review of the role of the microbiota–gut–brain axis in neurodegenerative diseases was recently published by Loh et al. [250].

In AD, alterations in the intestinal microbial population have been detected, with a reduction in SCFA-producing species and SCFA levels in subjects already in the preclinical phase of the disease compared to controls [271,272,273,274]. Furthermore, in patients with cerebral amyloidosis, a significant reduction in *Eubacterium rectale* and *Bacteroides fragilis* bacteria with anti-inflammatory characteristics was observed [275].

Diet can intervene and modify the microbiota–gut–brain axis, underlining the important role of food on human health. Specifically, DHA and EPA regulate the HPA axis by mitigating excessive cortisol production [276,277]. Furthermore, several authors have recently studied the influence of omega-3 PUFAs on the composition of the human intestinal microbiota and on the integrity of the intestinal barrier [278,279,280]. DHA and EPA supplementation positively modified the composition of the microbiota by promoting anti-inflammatory taxa such as *Bifidobacterium* and *Lactobacillus* [281,282] and reducing pro-inflammatory *Enterobacteriaceae* [283]. They also improved the levels of SCFA [284], which, after crossing the BBB, promoted microglial polarization towards an anti-inflammatory phenotype [285] and induced the expression of brain-derived neurotrophic factor (BDNF) and glial cell line-derived neurotrophic factor (GDNF), which are important for synaptic plasticity [286,287,288,289]. Furthermore, omega-3 PUFAs helped preserve the integrity of the intestinal barrier and the BBB [207].

Therefore, omega-3 fatty acid supplementation can be considered as a prebiotic strategy for a healthy intestinal microbiota and proper gut–brain communication.

### 4.9. Omega-3 Fatty Acids as miRNA Modulator

MiRNAs are small, noncoding RNA molecules composed of approximately 22 nucleotides that post-transcriptionally regulate gene expression by binding to complementary 3′ untranslated regions (3′-UTRs) of mRNAs, promoting their degradation or inhibiting their expression [290].

MiRNA-mediated epigenetic regulation in the etiopathogenesis of AD is a relatively new and highly interesting field of research. Growing evidence shows the involvement of miRNAs in several pathophysiological processes of AD, and the dysregulated expression of miRNAs in both the brain and blood of AD subjects [291,292,293,294,295,296,297].

Omega-3 PUFAs are increasingly emerging as important epigenetic regulators in aging and neurodegenerative diseases. Recently, some authors have demonstrated the ability of omega-3 PUFAs to alter the levels of certain miRNAs [298,299,300]. This is of great importance because, once again, it highlights how a correct diet can influence human health.

Ding et al. have demonstrated the neuroprotective effect of a diet enriched with omega-3 fatty acids on neurotoxicity induced by exposure to diethylhexyl phthalate (DEHP), a plasticizer used in many products that can cause cognitive impairment [301]. Omega-3 fatty acids improved hippocampal synapses and alleviated learning and memory deficits in mice, normalizing the levels of 14 miRNAs associated with synaptic density thickness that were altered by DEHP exposure [301].

Several studies have focused on the ability of PUFAs to influence inflammatory processes through the modulation of miRNA expression. In a rat model of inflammation, Zheng et al. reported a significant downregulation of rno-miR-19b-3p, rno-miR-146b-5p, and rno-miR-183-5p. A diet rich in omega-3 PUFAs appears to attenuate inflammatory responses by modulating the transcription of these miRNAs, ultimately leading to the reduced expression of multiple inflammation-related genes [300]. Intriguingly, miR-19b-3p and miR-146b-5p also appear to be dysregulated in the serum of AD patients [302,303].

Additionally, dietary supplementation with ω3-PUFAs inhibited neuroinflammation and improved cognitive function by regulating the expression of miR-107 in the brain of a mouse model of LPS-induced neuroinflammation [304].

Furthermore, D-series resolvins ameliorated inflammation by inhibiting NF-κB activation and decreasing the production of pro-inflammatory cytokines through the regulation of specific miRNAs [134,135,136].

An interesting study by Li et al. highlights the association between PUFA consumption, microbiota, and miRNAs in regulating obesity-associated cognitive impairment in older adults. In summary, PUFA intake can improve cognitive function, regulating the expression of specific miRNAs (hsa-miR-103a-3p, hsa-miR-107, hsa-miR-142-5p, and hsa-miR-144-3p) by affecting the diversity and abundance of the gut microbiota [305].

The neuroprotective action of DHA was recently shown by Hu et al. in a cellular model of AD [306]. DHA activated microglial autophagy and improved the pathological burden through the modulation of the miR-589-5p/toll-like receptor 4 (TLR4) axis, which is altered in AD patients [306].

To date, few studies have examined the impact of a PUFA-enriched diet on the regulation of miRNA expression in patients with MCI or AD. Therefore, further exploration of the role of omega-3 fatty acids (particularly DHA and EPA) in AD epigenetics is warranted, as current knowledge suggests that lifestyle-related changes in miRNAs could influence the efficacy of therapies and significantly impact the course of the disease.

## 5. What Do Clinical Studies Indicate?

The enormous neuroprotective potential of PUFAs, summarized in Table 1, has spawned numerous clinical trials seeking to verify their efficacy in countering the signs of AD, but despite the excellent premises, the effects of omega-3 fatty acid supplementation on cognitive functioning remain unclear, and the results are rather inconsistent.

Several clinical studies have shown a correlation between DHA levels and the risk of cognitive impairment. Low DHA values have been associated with a higher likelihood of developing dementia [307,308]. Consistently, in a study conducted by Conquer et al., patients with AD showed significantly reduced plasma concentrations of omega-3 PUFAs and DHA compared to controls, and this reduction was more marked in the advanced stage of the disease [309]. Meta-analysis studies conducted by Pan et al. [310] in 2015 and Zhang et al. [311] in 2016 confirmed these data.

Conversely, a Mendelian randomization analysis by Tomata et al. reported that PUFAs had no effect on the risk of developing AD [312]. A study by Quinn et al. also found no positive effect of DHA supplementation on cognitive decline in patients with mild to moderate AD [313], and several other studies have found no benefit following treatment with omega-3 PUFAs in elderly individuals with or without dementia [314,315,316,317].

A 12-month randomized, double-blind, placebo-controlled study in 36 elderly subjects with MCI reported improved memory following supplementation with DHA-concentrated fish oil [318]. These data were subsequently supported by a larger study conducted on 240 MCI patients treated with DHA (2 g/day) and placebo for 12 months in which not only a significant improvement in cognitive function was observed, but also a slowing of the progression of hippocampal atrophy [319]. Similar positive results for fish oil supplementation on cognitive functioning have been obtained in previous studies [320,321,322,323,324].

Kosti et al.’s systematic review and dose-response meta-analysis of observational and experimental studies on fish intake and the incidence of dementia reported that fish consumption of up to 2 portions per week was associated with a non-statistically significant 10% reduction in dementia risk and a 30% reduction in AD risk in later life, while further increasing consumption did not appear to provide substantial additional benefits [325].

Consistently, a recent meta-analysis of 58 randomized controlled clinical trials on the effects of omega-3 supplementation on cognitive function, conducted by Shahinfar et al., found that daily supplementation with amounts between 1000 and 2500 mg of omega-3 fatty acids was associated with significant improvements in attention, perception speed, language, primary memory, visuospatial function, and global cognitive ability, followed by a downward trend at higher doses. Furthermore, no adverse effects were reported among participants taking omega-3 supplements within this dosage range [326].

These data indicate the importance of the dosage and duration of treatment. It is therefore essential to identify the optimal range within which to maintain omega-3 fatty acid concentrations to achieve the maximum benefit.

An interesting finding concerns the effect of DHA supplementation in subjects carrying the apolipoprotein ε4 (APOE4) allele, the main genetic risk factor for the development of late-onset AD [327]. APOE protein influences the metabolism and transport of cholesterol and lipids in the brain, and APOE4-positive subjects with AD show lower brain DHA intake and a reduced DHA/AA ratio than those APOE4-negative with a greater loss of hippocampal volume [328]. It has been observed that APOE4 carriers with dementia do not respond to DHA supplementation, whereas ε4-negative subjects show a significantly lesser cognitive decline [329,330,331]. This suggests the importance of genetic factors in treatment response and may explain the variability of results observed in some clinical studies.

Promising results on the effects of high-dose omega-3 and omega-6 fatty acid supplementation, in combination with antioxidant vitamins, on cognitive function in older adults with MCI were obtained in a 6-month randomized, double-blind, placebo-controlled study [89,332]. These data were confirmed by a meta-analysis of randomized controlled trials on the effects of vitamins and PUFAs on cognitive function and memory in older adults with MCI [333].

Overall, these studies indicate a promising role for omega-3 fatty acids in the treatment of Alzheimer’s disease.

The heterogeneity of responses observed in these studies could be attributed to the different cognitive conditions of participants at enrollment, the genetic factors, the doses of omega-3 fatty acids administered, and the duration of treatment. Furthermore, it has been reported that the change observed with supplementation is greater the further baseline PUFA status deviates from the threshold of change [334,335]. Many studies do not report omega-3 levels at patient enrollment and/or at study completion, and since the observed effect may depend on significantly increased DHA levels from baseline after treatment, knowing blood levels of DHA and EPA before and after treatments is important to interpret, understand, and compare observed changes across studies [336].

Therefore, a standardization of studies is needed to more clearly define the efficacy of treatments, and a baseline omega-3 deficiency should be a prerequisite for demonstrating the efficacy of a treatment.

A detailed analysis of the critical issues of clinical studies was provided by von Schacky et al. [337].

## 6. Conclusions

A significant body of research supports the potential neuroprotective activity of omega-3 fatty acids against neurodegenerative diseases such as Alzheimer’s, primarily due to their anti-amyloidogenic, anti-inflammatory, antioxidant, and anti-apoptotic properties.

Studies conducted to date, albeit with several limitations, have demonstrated the significant impact of omega-3 PUFAs on brain health. However, several recent cohort studies have shown that the only observed positive effects involve a delay in cognitive decline in MCI patients [318,321,323,338], suggesting that a diet enriched with omega-3 PUFAs could help prevent AD in at-risk individuals or delay progression to AD in individuals with MCI, but becomes less effective once the disease has become established [144,339]. However, larger multicenter studies are needed to establish effective clinical benefits.

Individual genetic variants, combined with lifestyle (diet, physical activity, sleep, environment) and the composition of the gut microbiota, may play a significant role in treatment efficacy, increasingly leading to a multi-targeted and personalized therapeutic approach. Moreover, aging can alter the absorption and conversion of omega-3 PUFA precursors into active metabolites.

Recently, it has been shown that DHA in its esterified form (lysoPC-DHA) is better incorporated into the brain than the non-esterified form, and mice orally treated with lysoPC-DHA showed improved memory and cognitive function compared to the same amount of free DHA [33,340,341]. Furthermore, Otaegui et al. demonstrated that intranasal treatment with a DHA-enriched nanoemulsion, designed to protect DHA from oxidation, in a transgenic mouse model of AD (J20 mice) reduced amyloid levels, oxidative stress, neuroinflammation in brain tissues, and improved spatial working memory [342]. Therefore, attention should also be paid to the type of formulation and the brain bioavailability [343].

Further studies are thus encouraged to deepen understanding of the mechanisms of action, confirm the therapeutic potential of omega-3 PUFAs in neurodegenerative diseases such as AD, investigate their synergistic action with equally effective neuroprotective molecules, study new formulations capable of improving brain bioavailability, and analyze whether there is room for improving intervention strategies by including them in existing therapeutic regimens.

## Figures and Tables

**Table 1:** Summary of key neuroprotective potential effects of omega-3 fatty acids in AD and related animal studies.

Mechanism	Target	Study Design	Main Findings
Aβ accumulation	PS1	3-month-olds 3xTg-AD mice fed with 1.3 g DHA/100 g feed and a 1:1 ratio of n-6 to n-3 fatty acids versus a control diet for 3–9 months.	Reduction of brain Aβ level and PS1 activity [109].
	BACE1	5-month-old APP/PS1 mice, treated with DHA (400 mg/kg) orally once daily for 2 months.	Reduction of Aβ plaque deposition and BACE1 expression in the brain and improved learning and memory functions [110].
Tau hyperphosphorylation	JNK	5-month-old 3xTg-AD transgenic mice fed a high-fat diet supplemented with fish oil (calculated to provide 0.6% DHA) for 4 months.	Reduction of JNK, IRS-1, and tau phosphorylation associated with improved performance in the Y-maze [118].
		9-month-old SAMP8 mice, orally treated with DHA (200 mg/kg) for 20 days.	Reduction in p-JNK levels in the hippocampus associated with a decrease in tau protein hyperphosphorylation and improvement of cognitive functions [119].
Neuroinflammation	SIRT1/HMGB1/NF-κB	Traumatic brain injury model rats treated with n-3 PUFA (2 mL/kg) intraperitoneally once daily for 7 days.	Suppression of pro-inflammatory cytokines and promotion of anti-inflammatory phenotype in microglial cells through SIRT1-mediated deacetylation of the HMGB1/NF-κB pathway, resulting in neuroprotective effects from experimental traumatic brain injury [124].
	PI3K/Akt/ mTOR	3–4-week-old Wistar rats with NA-STZ-induced type 2 diabetes mellitus, treated with RvD1 (60 ng/animal) for 5 days.	Reduction of pro-inflammatory cytokine production, restoration of LXA4 and BDNF levels, and improvement of neuronal survival through modulation of the PI3k-Akt-mTOR pathway in the brain [139].
	PI3K/Akt and p38MAPK	3–4-month-old C57BL/6 mice with stereotaxic intrahippocampal injection of Aβ42 and treatment with 1 μL of MaR1 solvent.	Improvement of cognitive decline and neuronal survival by enhancing autophagy and inhibiting inflammation and apoptosis pathways [141].
Oxidative stress	Nrf2/HO-1	C57BL/6 mice subjected to 60 min of MCAO, fed for 6 weeks with a PUFA-enriched diet (DHA + EPA from 0.34 to 1.5%, n-6:n-3 PUFA ratio from 5:1 to 1:1).	Neuroprotection against ischemic brain injury through enhanced Nrf2 activation and upregulation of HO-1 [167].
		7-week-old Wistar rats treated intragastrically with DHA (370, 555, or 740 mg/kg per day) 30 min after head injury.	Neuroprotection against traumatic brain injury (TBI), improvement of neurological and cognitive functions, and reduction of oxidative stress by activation of Nrf2 and increased expression of downstream factors NQO-1 and HO-1 [172].
Ferroptosis	RORα and Nrf2 signaling pathways	8–10-week-old BALB/c mice subjected to whole-brain irradiation, treated intraperitoneally with MaR1 1–3 mg/kg for 3 days.	Improvement of neurological function and ferroptosis associated with radiation-induced brain damage in mice through the RORα and Nrf2 signaling pathways, with a reduction in COX2 and an increase in GPX4 [192].
Mitochondrial Dysfunction	Pink1/Parkin mitophagy	6-week-old MCAO mouse model treated intraperitoneally with DHA (10 mg/kg) once a day for 3 days.	Increase of Pink1/Parkin-mediated mitophagy, enhancement of mitochondrial metabolic capacity, and improved neurological function after stroke [204].
Insulin Resistance	SIRT-1	C57BL/6- HFD (9 months) for 20 weeks treated with DHA (100 mg/kg, twice a week) orally for 8 weeks.	Improvement of diet-induced obesity and insulin resistance, antiangiogenic effect, and increased SIRT1 expression in adipose tissue [213].
Glucose metabolism	GLUT1	Gray mouse lemurs at the age of 23 ± 4 months supplemented for 12 months with 6 mg EPA and 30 mg DHA.	Increased glucose uptake and utilization in the primate brain and improved performance in the Barnes maze [227].
Cerebral clearance system	AQP4	Fat-1 mice (8–12 weeks) treated daily with 30 mg/kg fish oil (52.4% DHA) orally for 3 weeks.	Enhanced interstitial clearance of Aβ from the brain in an AQP4-dependent manner and protection from Aβ-induced neuronal damage [113].
		6–8 weeks old C57BL/6 mice treated with omega-3 fish oil (15 g/kg) for 2 months prior to TBI induction.	Enhanced glymphatic clearance of Aβ and prevention of BBB disruption after induction of traumatic brain injury [241].
	LRP-1	APP/PS1 4-month-old mice treated with 50 μL fish oil (containing 13 μM EPA and 99 μM DHA) daily for 4 weeks.	Increased LRP-1 expression levels in brain capillary endothelium, enhanced Aβ clearance, and improved neuroinflammation levels [247].
MiRNA	miR-19b-3p, miR-146b-5p, and miR-183-5p	3-week-old Wistar rats treated with omega-3 PUFAs 10 μL/100 g/day for 16 weeks.	Reduction of inflammation by regulating the transcription of related miRNAs [300].
	PSD-associated miRNAs	4-week-old mice treated with omega-3 fatty acids (150 mg/kg) for 8 weeks.	Protection against DEHP-induced cognitive impairment and improvement of synaptic structure in the hippocampus by regulating the expression of PSD-associated miRNAs [301].
	miR-107/PIEZO1/NFκB p65	Mouse model of LPS-induced neuroinflammation (6 weeks old) treated with ω3-PUFA (2 mL/kg).	Increased miR-107 expression, reduced PIEZO1/NFκB p65 pro-inflammatory pathway, ameliorated LPS-induced neuroinflammation and cognitive impairment in mice [304].

## Data Availability

Not applicable. No new data were created or analyzed in this study.

## Abbreviations

The following abbreviations are used in this manuscript:

AD	Alzheimer’s disease
Aβ	Amyloid-β
PUFA	Polyunsaturated fatty acids
EPA	Eicosapentaenoic acid
DHA	Docosahexaenoic acid
MCI	Mild cognitive impairment
NFT	Neurofibrillary tangles
CSF	Cerebrospinal fluid
GLA	γ-linolenic acid
AFA	Aphanizomenon flos-aquae
ALA	α-linolenic acid
AA	Arachidonic acid
SFA	Saturated fatty acids
MUFA	Monounsaturated fatty acids
ATP	Adenosine triphosphate
CNS	Central nervous system
FADS	Fatty acid desaturase
ELO	Fatty acid elongases
MFSD2a	Major facilitator superfamily domain-containing protein 2a
BBB	Blood–brain barrier
FABP5	Fatty acid-binding protein 5
FATP	Fatty acid transport proteins
ACSL	Long-chain acyl-CoA synthetase
PE	Phosphatidylethanolamine
PC	Phosphatidylcholine
PD	Parkinson’s disease
PPAR	Peroxisome proliferator-activated receptors
NF-κB	Nuclear factor κB
SREBP	Sterol regulatory element-binding proteins
LXR	Liver X receptors
TGF-β	Transforming growth factor-β
SPM	Specialized pro-resolving mediators
LOX	Lipoxygenase
COX	Cyclooxygenase
PS	Phosphatidylserine
PI	Phosphatidylinositol
PSD-95	Postsynaptic Density Protein 95
APP	Amyloid precursor protein
IR	Insulin resistance
BACE1	Beta-secretase 1
IDE	Insulin-degrading enzyme
AQP4	Aquaporin-4
GSK-3β	Glycogen synthase kinase-3 beta
JNK	c-Jun N-terminal kinase
TNFα	Tumor necrosis factor alpha
IL-1β	Interleukin-1 beta
MAPK	P38 mitogen-activated protein kinase
NPD1	Neuroprotection D1
RvD	D-series resolvins
RvE	E-series resolvins
Mar	Maresin
GPCR	G protein-coupled receptors
ChemR23	Chemokine-like Receptor
ROS	Reactive oxygen species
HO-1	Heme oxygenase
GPX4	Glutathione peroxidase 4
NAD	Nicotinamide adenine dinucleotide
Nqo1	Quinone oxidoreductase 1
ABAD	Amyloid-binding alcohol dehydrogenase
TFAM	Mitochondrial transcription factor A
TFB2M	Mitochondrial transcription factor B2
Nrf	Nuclear respiratory factor
ERRα	Estrogen-related receptor alpha
PGC-1α	PPARG coactivator 1 alpha
Mfn	Mitofusin
Opa-1	Optic atrophy protein 1
GLUT	Glucose transporter
LRP-1	Lipoprotein receptor-related protein 1
ENS	Enteric nervous system
HPA	Hypothalamic–pituitary–adrenal
SCFA	Short-chain fatty acids
GABA	γ-aminobutyric acid
BDNF	Brain-derived neurotrophic factor
GDNF	Glial cell line-derived neurotrophic factor
DEHP	Diethylhexyl phthalate
TLR4	Toll-like receptor 4
APOE	Apolipoprotein E
